# Exploring Silent Speech Interfaces Based on Frequency-Modulated Continuous-Wave Radar

**DOI:** 10.3390/s22020649

**Published:** 2022-01-14

**Authors:** David Ferreira, Samuel Silva, Francisco Curado, António Teixeira

**Affiliations:** 1 Department of Electronics, Telecommunications & Informatics, University of Aveiro, 3810-193 Aveiro, Portugal; fcurado@ua.pt (F.C.); ajst@ua.pt (A.T.); 2 Institute of Electronics and Informatics Engineering of Aveiro (IEETA), 3810-193 Aveiro, Portugal

**Keywords:** silent speech, continuous-wave radar, European Portuguese, machine learning

## Abstract

Speech is our most natural and efficient form of communication and offers a strong potential to improve how we interact with machines. However, speech communication can sometimes be limited by environmental (e.g., ambient noise), contextual (e.g., need for privacy), or health conditions (e.g., laryngectomy), preventing the consideration of audible speech. In this regard, silent speech interfaces (SSI) have been proposed as an alternative, considering technologies that do not require the production of acoustic signals (e.g., electromyography and video). Unfortunately, despite their plentitude, many still face limitations regarding their everyday use, e.g., being intrusive, non-portable, or raising technical (e.g., lighting conditions for video) or privacy concerns. In line with this necessity, this article explores the consideration of contactless continuous-wave radar to assess its potential for SSI development. A corpus of 13 European Portuguese words was acquired for four speakers and three of them enrolled in a second acquisition session, three months later. Regarding the speaker-dependent models, trained and tested with data from each speaker while using 5-fold cross-validation, average accuracies of 84.50% and 88.00% were respectively obtained from Bagging (BAG) and Linear Regression (LR) classifiers, respectively. Additionally, recognition accuracies of 81.79% and 81.80% were also, respectively, achieved for the session and speaker-independent experiments, establishing promising grounds for further exploring this technology towards silent speech recognition.

## 1. Introduction

Speech is a natural and efficient form of human communication, and, as such, the research on speech technologies that can foster its use in domains such as Human–Computer Interaction (HCI) is highly relevant. While Automatic Speech Recognition (ASR) is commonly used in HCI environments, as in the case of Amazon’s Alexa and Apple’s Siri [1], there are still some scenarios that cannot take the most out of speech interaction, including situations where privacy is needed, environmental noise is present, silence is required, or in the most extreme cases, when health conditions incapacitate speakers to produce acoustic signals.

To tackle such scenarios, Silent Speech Interfaces (SSI) emerged as a possible alternative to consider, consisting of the process of speech communication in the absence of an audible/intelligible acoustic signal [2]. As speech production is a complex motor process, which starts in the brain and ends with respiratory, laryngeal, and articulatory motion, each step of its production process can be explored and physiologically measured through specialized sensors and methods to potentially infer what the speaker is trying to say without relying on the acoustic signal [3,4].

Although there are already a large set of proposed sensors and technologies for silent speech recognition, most of them have characteristics that limit their use in everyday life, e.g., by being intrusive, non-portable, affected by noise, user-dependent, or just not affordable [5,6,7,8].

In light of these challenges, it is important to explore novel and improved technologies that might bring SSI to a wider variety of scenarios and users. To this end, frequency-modulated continuous wave (FMCW) radar technology, already used in a wide variety of scenarios including the automotive industry [9,10] and service robots [11,12], emerges as a possible candidate to tackle some of these issues given its non-invasiveness, non-intrusiveness, portability, and independence of ambient lighting. Furthermore, recent evolutions have made it less costly and easily available commercially, making radar technology appear in many of our daily environments. The recent market launch of the first mobile phone with radar (Google pixel 4; 2019) dedicated to proximity detection and identification of manual commands from the user without direct contact with the device, points towards the vulgarization of this technology and opens up prospects of its future exploitation for novel applications. In this regard, the exploration of a technology that might already be present to bring SSI capabilities to the environment is a promising path to follow. However, while a strong potential can be anticipated, given these perceived advantages, radar technology has yet to prove its mettle for silent speech applications.

In previous work, the authors performed a preliminary study exploring the capabilities of contactless radar-based technology for silent speech interfaces [13]. The achieved results, obtained considering a corpus of 13 European Portuguese words and three speakers, demonstrated good overall performance establishing the feasibility of the proposed approach and yielding promising grounds for additional research. In this context, the main goal of the work presented here is to expand on previous work regarding radar-based SSI and contribute to the body of work in the field by (a) expanding the number of considered speakers, in regards to our previous work, from three to four; (b) assessing session independence by considering data obtained for the same speakers in two independent acquisition sessions; and (c) exploring speaker-independent performance.

The remainder of this document is structured as follows. Section 2 presents a brief overview on related work regarding non-invasive SSI, also covering previous research on SSIs for European Portuguese. Section 3 describes the adopted methods, from environment and acquisition settings to data exploration, feature extraction, and classification approaches. Section 4 reports the results for all the performed research experiments (i.e., per-speaker, intra-speaker, and inter-speaker). In Section 5, these are further analyzed and discussed. Finally, Section 6 presents some concluding remarks and ideas for further advancing this work.

## 2. Related Work on SSI

A distinguishing element of SSIs is speech recognition beyond the acoustic signal, exploring other biosignals associated with the different stages of the speech production process [2,4]. From brain waves to the visual aspects of speech, several approaches have been, and continue to be, proposed towards silent speech recognition (SSR). While relevant work also exists for invasive technologies, the overview provided in what follows focuses on non-invasive methods, as they are in line with our goals. Along with the presented overview, Table 1 is also presented, encompassing attained results from different considered technologies in the existing literature in the most recent years. Apart from researches that mainly focused on classification purposes, several others that studied the possibility of achieving session and speaker independence were also included, as they are topics incident on this work that will be subject to further exploration. Regarding the literature review process, Google Scholar was the search engine resorted to given its vast scope of scholarly literature.

In SSI development research, surface EMG (sEMG) is the most commonly used technology as it is easy to apply and is less prone to raise ethical concerns for volunteers [14,15]. Recent work has privileged the evolution and consideration of increasingly imperceptible and highly flexible sEMG electrodes (see, e.g., in [16,17,18,19,20]), and notable results include those of Liu et al. [16] and Dong et al. [15], where accuracies greater than 80% (for a vocabulary of six words) and 70.00% (for a vocabulary of three words) were, respectively, achieved. Nevertheless, there is still a high data variability between sessions due to the participants’ skin impedance [21].

Non-audible murmur (NAM) microphones are another technology widely used in SSI development to capture and record murmured speech and other smooth vocal productions resultant from the acoustic output. Recognition rates of nearly 70.00% were achieved in a total of 21 tested utterances [22]. However, NAM is highly user-dependent due to participants’ physiological differences, and its acquired waveforms typically lack in quality and intelligibility, consisting of open challenges that still need addressing [22].

Electroencephalography (EEG) enables measuring electrical brain signals in a non-invasive way. Recent studies (see, e.g., in [23,24]) attained accuracy rates around the 70% mark for corpora of five syllables and six imagined sounds (i.e., non-articulated, just imagined by the subjects, corresponding to the vocalization of the five vowels, a/i/u/e/o, and mute). Although allowing visualization of the activation of the different brain areas associated with speech production, this technology is highly sensitive to noise, and its recognition rates are user-dependent.

**Table 1:** Notable recent studies tackling silent speech recognition and the outcomes regarding speaker and session independence. Apart from stating the considered technology, for each study, its publication year and acquisition corpus (when applicable) are also presented.

	Tech.	Year	Corpus	Accuracy	Inter-Speaker	Intra-Speaker
Ma et al. [25]	sEMG	2019	10 words	72.00%	-	-
Rameau et al. [26]	sEMG	2020	2 isolated words	86.40%	-	-
Prorokovic et al. [27]	sEMG	2019	-	-	-	Lower WER than conventional methods
Meltzner et al. [14]	sEMG	2018	65 words 1200 word sequences	91.40% MFCC 94.20% with grammar models	Thousands of recorded hours are required from diverse population	-
Wand et al. [28]	sEMG	2018	-	-	-	Lower WER than conventional methods
Fernandes et al. [29]	sEMG	2019	2 isolated words	84.00% for 2 words	-	-
Liu et al. [16]	sEMG	2020	1 set of 5 words 1 set of 6 words	89.60% for set 1 92.70% for set 2	-	-
Kapur et al. [20]	sEMG	2018	15 words	92.01%	Future Work	-
Dong et al. [15]	sEMG	2019	3 words	71.70%	-	-
Shah et al. [22]	NAM	2018	21 utterances	64.33%	-	-
Sarmiento et al. [23]	EEG	2019	5 syllables	69.73% to 72.67%	-	-
Morooka et al. [24]	EEG	2018	6 sounds	79.70%	-	-
Chen et al. [30]	US	2018	-	-	Future Work	-
Zhao et al. [31]	US	2019	-	-	Future Work	-
Gosztolya et al. [32]	US	2019	-	-	Future Work	Future Work
Kimura et al. [33]	US	2019	4 commands	65.00%	-	-
Csapó et al. [34]	US	2019	9 sentences	78.84%	-	-
Sun et al. [35]	VID	2018	20 commands limited to usage context with lip exaggeration	98.90%	95.40%	-
Vougioukas et al. [36]	VID	2019	GRID database	73.40%	59.50%	-
Uttam et al. [37]	VID	2019	Oulu VS2 database	-	PESQ scores similar to speaker-dependent models	
Petridis et al. [38]	VID	2018	10 digits 10 phrases	-	70.50% 70.80%	-
Birkholz et al. [39]	UWB	2018	25 phonemes	89.00%	-	-
Dash et al. [40]	MEG	2019	5 phrases	79.93% imagination	22.10% without adapt. 55.08% with adapt.	-

sEMG = Surface Electromyography; NAM = Non-Audible Murmur; US = Ultrasound; VID = Video; UWB = Ultra-Wideband; MEG = Magnetoencephalography

Ultrasound (US) imaging is another technology widely considered in SSI research, as it allows observing tongue movement sequences during the speech production process. Some recent works include those of Chen et al. [30] and Xu et al. [41], in which, respectively, a new technique for representing speech articulation resorting to an ultrasound-driven finite element model of the tongue is presented, and a novel sequential feature extraction approach for SSI systems is explored. Considering US studies, recognition results are typically disregarded, as they are mainly focused on synthesizing speech that is further subject to subjective assessment regarding how natural they sound [33,34]. Nevertheless, while US images yield good spatial and temporal resolutions, the images have relatively low quality due to the presence of speckle noise [42].

Video imaging can be used to capture visible speech articulators. By resorting to different models and algorithms, which allow extracting the articulators of interest from each frame, it is possible to obtain accuracy rates as high as 87.00% for a corpus of eight consonants [43] and 98.00% for 20 commands limited to usage context with slight over-articulation to increase the extent of lip movement [35]. Although generally being low-cost, this technology is susceptible to raise privacy concerns and are typically strongly affected by ambient illumination.

Regarding contactless radar-based silent speech recognition, and to the best of our knowledge, not much has yet been explored apart from a recent work by Shin et al. [8]. In this study, a recognition rate of 85.00% was reported for a corpus comprising 10 isolated words considering a dynamic time warping (DTW) approach. However, as stated by the authors, some limitations resided in the fact that distance and correlation amplitude were the only considered features, and that there was recognition degradation due to slight head movements of the participants throughout the acquisition sessions, something that would require additional methods to mitigate.

Concerning SSI for European Portuguese (EP), several technologies have been researched (e.g., EEG, sEMG, Ultrasonic Doppler (UD), Video, and Depth), also including multimodal approaches [7]. Freitas et al. [44] proposed Visual Speech Recognition (VSR) and Acoustic Doppler Sensors (ADS) for silent speech recognition, resorting to Dynamic Time Warping (DTW), achieving a 91.40% accuracy rate. Later, in 2013, the same author [45] selected four non-invasive modalities (Visual data from Video and Depth, sEMG, and UD) and proposed a system that explores their synchronous combination into a multimodal SSI. The same corpus as the one explored in this article was considered, and DTW and KNN were used. It was verified that the combination of multiple modalities presented a better recognition performance (93.80%), while subsets of the modalities produced lower results, such as 71.40% for the Video and Depth combination. In a more recent work, Teixeira et al. [46] proposed a VSR approach to enable real-time control of a media player, having achieved an accuracy of 81.30% for a corpus comprising eight control commands.

## 3. Method

In line with our research goals, and regarding its acquisition settings, an approach was established in which there was a compromise between keeping some aspects closer to real scenarios (e.g., no chin rest during acquisitions) and establishing some controlled conditions (e.g., frontal head orientation and fixed approximate distance from the radar). Such considerations, while not compromising the study’s central purpose, should reduce, at this stage, some of the complexity of its data acquisition and post-processing phases.

**Figure 1:**
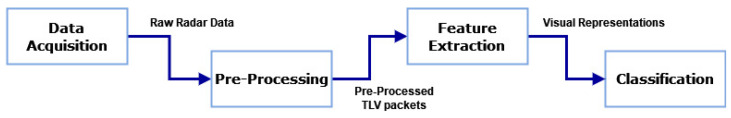
Acquisition and classification pipeline. From left to right, the respective processing steps are data acquisition, preprocessing, feature extraction, and classification.

In this section, all steps that are inherent to this project’s methodology are described and the key stages are illustrated in Figure 1.

### 3.1. Experimental Setup

The board considered for this investigation was the AWR1642BOOST-EVM from Texas Instruments, Dalas, TX, USA, an evaluation board for the AWR1642 FMCW radar sensor. This board is currently used at our research institute for a plethora of different purposes, ranging from robot navigation and human detection [47] to biosignal measurement [48]. The room selected for the acquisition sessions was free from any moving objects other than the participant, ensuring that no interference would negatively impact the acquired data.

In addition to the room settings, it was also established that, throughout the acquisition sessions, the participants would directly face the radar and sit at an approximate distance of 15 cm from it, a similar setting to how speakers are positioned in front of a tabletop microphone. However, and as we were aiming for reduced intrusiveness, we opted not to fix the position of the participants’ heads, simply instructing them, without enforcing it, to try and keep the same relative position towards the radar.

### 3.2. Data Acquisition

For the data acquisition sessions, we resorted to Texas Instruments’ DemoVisualizer application, a software that enables radar configuration, data acquisition, and data visualization. DemoVisualizer enabled testing different radar configurations while focusing on the acquisition aspects that most suited the particular research experiments. As previously explained, we privileged a less fixed head position, and this, as observed by the authors of [8], might yield added challenges in using distance to the radar as the data considered for recognition. Therefore, we opted for a configuration that prioritized the best possible velocity resolution, envisaging the acquisition of the participants’ facial velocity dispersion patterns while they produced speech.

**Figure 2:**
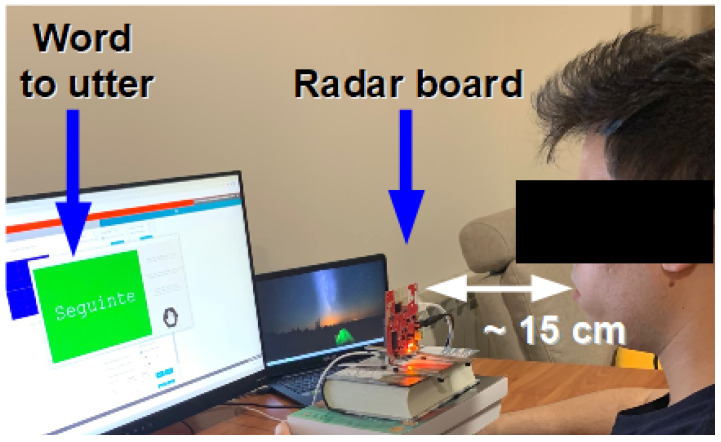
Radar setup for the data acquisition sessions. The participant is seated in front of the radar board while a monitor continuously displays the words to be uttered, turning the background green whenever the participant is asked to speak.

To automatically manage the data acquisition process, custom software was designed and developed. The participants were placed in front of the radar board, at approximately 15 cm from it (Figure 2). An LCD display, adequately positioned in the participant’s line of sight, provided information about the word to be uttered. After signing the informed consent, speakers were asked to speak at a normal rhythm, and, subsequently, the acquisition procedure started. For each trial, a random corpus word would appear on the LCD, and a beep (along with a change in color of the screen) would signal that the speaker should utter it. After the beep, the acquisition software recorded radar data for two seconds.

It is also important to mention that, in addition to the first acquisition session, the realization of a second session was scheduled for three months later for those participants that could perform it to allow studying intra-speaker variability.

### 3.3. Corpus

Considering the team’s body of work on Ambient Assisted Living (AAL) and its previous work in SSI research for these contexts, we adopted a previously considered corpus [45,49], presented in Table 2, containing a total of thirteen EP words. The words aggregated in the chosen corpus are mostly command instructions typically used in AAL contexts, such as when interacting with personal assistants (e.g., asking the personal assistant to turn something on, contact someone, or help in any way it can).

Four participants, all native EP speakers, enrolled in the data acquisition sessions: (a) one of the authors, an Engineering Ph.D. student, 26 years old, male; (b) a 24 years old female Psychologist; (c) a 50 years old female real estate manager; and (d) a 22 years old female Physiotherapist. Two of the speakers (one of them, Speaker 1, the first author, and Speaker 4) were asked to try and keep a more consistent speaking pattern throughout each acquisition session. This would inform a best-case scenario where a prospective user would be informed to be consistent in uttering the commands compared with speakers without such information.

For each of the 13 words present in the corpus, 60 validated repetitions were considered per participant. The validation stage mainly ensured the removal of the recordings in which no usable data was produced (e.g., participant missing the recording slot or data recording error).

### 3.4. Preprocessing

The preprocessing phase is mainly responsible for analyzing the produced data for each participant and adequately annotating each file with its corresponding class name and trial number. Besides organizing the data to a format convenient for the subsequent feature extraction phase, it also ensures the removal of any data inadequately acquired. The main difference between this processing step and the validation stage is that, while the validation stage consists of an empirical process where missed time slots or mispronunciations are removed through the observation of visual representations, this step removes the files at the earliest stage possible, verifying if the files, as soon as they are acquired by the radar board, are either corrupted or badly organized.

### 3.5. Feature Extraction

After the acquisition sessions, it was necessary to explore the data and define a set of features to be tested and used for classification. During data acquisition, the signals received by the RF front-end of the board are digitized and preprocessed by the built-in ADC and DSP, respectively, and the raw data thus obtained are assembled into several tag-length-value (TLV) packets. To process these packets, a parser was developed in Matlab to extract all the detected objects’ relevant information (i.e., their Cartesian coordinates (X, Y, Z) and relative velocities expressed in the radar frame of reference). These data include the entire point cloud detected in the radar FoV, over the time acquisition windows, from which distinct subsets of points are clustered and associated, in real-time, by the firmware of the board, to represent different objects, or parts of a body, with dissimilar velocity measures. However, besides including static and moving objects, these readings may also contain “fake” target detections that often result from multiple reflections on walls or other surfaces, which led us to define that all data beyond 30 cm from the radar board would be filtered and excluded.

**Figure 3:**
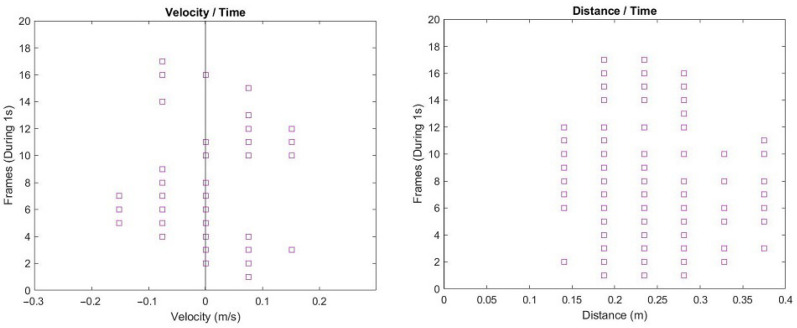
Illustrative visual representations for the data corresponding to one acquisition of the word “Ajuda” (Help): velocity dispersion pattern (**left**) and distance variation (**right**) over 1 second of acquisition frames (along the vertical axis). For this case study, although distance variations were acquired and presented, only the velocity representations were considered for model training and subsequent classification.

Regarding the visualization of the acquired data, for each word instance, two representations were created, one depicting distance variations over time and another the velocities dispersion over time (Figure 3).

Although distance information could potentially provide pertinent data for classification purposes, in this work, they were disregarded, given the verification that slight distance differences between recordings of the same word would produce substantially different representations. That is especially true in this specific case, as no chin rest was used throughout the acquisition sessions. While additional postprocessing could help minimize this issue [8], such consideration was left for future work, and we ultimately opted for exploring the dispersion of velocity data associated with the users’ facial motions.

#### Classifier Training and Testing

For testing the different classification approaches, the velocity dispersion data (as depicted in Figure 3) for each word instance (words being the classes) were provided to the machine learning algorithms. Additionally, and towards understanding the impact that different classifiers could have in the classification outcomes, several that have been commonly used in works pertaining SSI development and similar classification tasks were considered: Random Forests (RF), Linear Discriminant Analysis (LDA), Linear Regression (LR), Support Vector Machine (SVM), and Bagging (BAG). To implement them, Scikit-learning library was used with the different configuration parameters set to their default values. Regarding the classification process, a 5-fold cross-validation approach was adopted due to the limited size of the acquired data, ensuring that every observation from the original dataset had the chance of appearing in both training and testing. Finally, to assess the performance of the different classifiers, and due to the acquired dataset being class-balanced (i.e., no disparity between the number of instances belonging to each class), the Accuracy metric was adopted.

## 4. Results

As already mentioned, in this study, three experiments were conducted towards validating and assessing FMCW radar-based technology’s silent speech recognition capabilities. Although the main objective was to understand if good classification results could be achieved, understanding the possibility of creating session-independent and speaker-independent models was also pivotal. This section is responsible for presenting and describing the achieved results.

### 4.1. Per-Session Speaker Performance

The first research experience of this study consisted of assessing FMCW radar technology’s silent speech recognition capabilities. For this, the data acquired from both conducted acquisition sessions were considered. It is, however, worth mentioning that, although the initial plan was for all four speakers to participate in both sessions, one of them could not make it to the second due to personal life complications. In light of this circumstance, Table 3 presents the recognition results for the participants acquired on both sessions while excluding Speaker 3 from the recognition of the second session’s data. In this experience, the classification process was pretty straightforward, as the models were independently created and tested with the data from each speaker per acquisition session.

**Figure 4:**
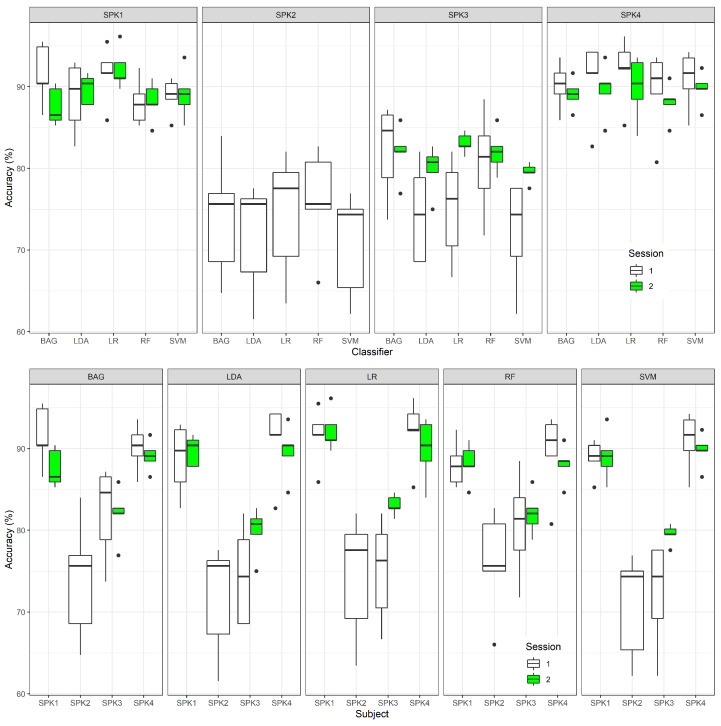
Boxplot representations depicting the average classification accuracies obtained for each acquisition session: average accuracies per classifier (**top**) and average accuracies per speaker (**bottom**). Speaker 2 only recorded one session.

Through a careful analysis of the summarized classification results presented in Table 3, and part displayed in graphical form in Figure 4, it is possible to verify that all mean accuracy values across all participants, for all different classifiers, were superior to 80.00%. For session 1, the best mean accuracy value was obtained from the BAG classifier (M=84.50; SD=8.70), while for session 2, the best mean accuracy value was obtained from the LR classifier (M=88.3;SD=4.70). LR, however, produced the maximum accuracy values of 96.10% for a specific k-fold iteration on both conducted sessions. The classifiers which attained the lowest mean accuracy values were SVM (M=80.70;SD=10.50) for session 1, and RF (M=86.00;SD=3.70) for session 2.

Regarding the accuracy values obtained for each speaker and classifier, it is possible to verify that, for Speaker 1, both BAG and LR classifiers achieved the highest mean accuracy values for session 1 (91.50%), while, for session 2, LR produced the highest value (92.20%), for Speaker 2, which only enrolled in one of the acquisition sessions, the RF classifier presented the highest mean accuracy value (76.00%), for Speaker 3, the BAG classifier produced the highest mean accuracy value for session 1 (82.20%), while, for session 2, LR classifier produced the highest value (83.10%), and, for Speaker 4, LR produced the highest mean accuracy values for both session 1 (92.20%) and 2 (89.80%).

Considering the average accuracy results obtained from all classifiers, for all speakers, as depicted in Figure 4, it is possible to verify that Speaker 1 and Speaker 4 presented higher accuracy values than the remaining, which, in turn, obtained results similar between themselves.

**Figure 5:**
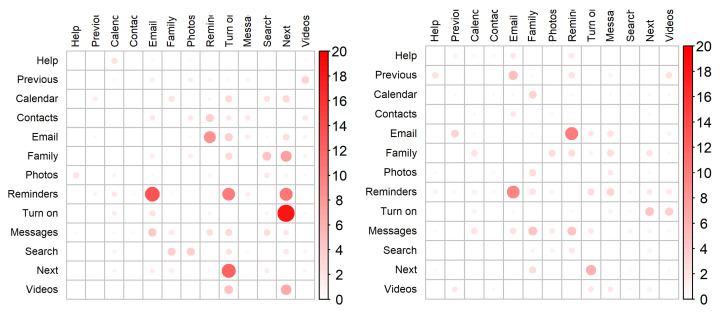
Confusion matrices for the best classifiers in each session illustrating the average recognition results across all participants. BAG classifier was considered for the first session (**left**), while LR classifier was considered for the second (**right**). The matrix rows represent the word instances submitted for recognition, while its columns represent the corresponding recognized words.

To further understand the recognition results and identify the words that were most commonly mistaken, thus, negatively contributing to the accuracy outcomes, two Confusion Matrices were created (Figure 5). These matrices depict the average accuracy classifications across all participants while considering the best classifier from each session (i.e., BAG for the first session and LR for the second). By analyzing the first session’s confusion matrix, it is possible to verify that the results were considerably worse for the words “Lembretes” (Reminders) and “Ligar” (Turn on) (with a correspondingly average accuracy, across all speakers, of 62.00% and 76.00%), typically being erroneously classified as “Email” and “Seguinte” (Next), respectively. As per the second session’s confusion matrix, the only classification result that scored below 80.00% was the one corresponding to the word “Lembretes” (Reminders), having achieved an average accuracy of 74.00%, being, just like in the first session, typically confused with the word “Email”.

### 4.2. Session-Independence Performance

The possibility of achieving session-independent models is highly desirable across the several technologies considered for SSR purposes [27,28,32]. The main factor for such a necessity is that, for embracing such technologies in daily scenarios, it is mandatory that different usages, even if considerably distant between different points in time, do not deteriorate the recognition results due to slight variability in the acquired data [27]. One example of a technology that severely suffers from such changes in the captured data across different sessions is sEMG, as removing and reattaching the electrodes in-between sessions causes variations in the recorded EMG signals [27,28].

As already mentioned, two acquisition sessions were performed for this research project, being its central purpose to, while considering the same corpus and validated word repetitions, capture a dataset equal to the first session’s one (besides the data acquired for the missing participant, Speaker 2). Although contributing towards all research experiences conducted in this study, its main intent was to allow performing this specific experiment, as it would allow understanding to what extent the creation of session-independent models is made possible by this technology.

After both datasets were acquired, the data belonging to the first session (session 1) was used for training, while the data from the second session (session 2) was considered for testing. The obtained recognition results are depicted in Table 4. In general, it is possible to verify that Speaker 1 obtained higher recognition results overall, being the best accuracy value obtained from the BAG classifier (M=81.79). For Speaker 3, RF classifier produced the best accuracy value of 67.95% and, for Speaker 4, BAG classifier produced the best accuracy value of 71.79%.

Regarding the relative variance values between the session-independent and session-dependent models, per speaker, it is possible to verify that Speaker 4 was the one that presented a higher variability, having a relative variance, for the LR classifier, of 28.87% for the first session’s data, and 26.47% for the second. Regarding Speaker 1, its session-independent model presented a maximum relative variance of 14.00% with RF classifier for session 1 and 14.32%, with LDA classifier, for session 1. For Speaker 3, its higher variances were 16.43% and 16.13%, both produced with the BAG classifier, for session 1 and 2 models, respectively.

### 4.3. Speaker-Independence Performance

Towards exploring and assessing FMCW radar-based technology’s capabilities regarding the creation of speaker-independent models, a third research experiment ensued. In this experiment, recognition models were created with data belonging to several speakers, i.e., each speaker’s data was left out for testing while the models were trained with the data belonging to the remaining speakers (n-1 models). The purpose of such consideration was to maximize the data from training, trying to achieve a more generalized recognition model, and approximate the case in which one intends to make a system that, after being trained, can be used by someone without the need to retrain the model with additional data.

The summarized classification results for the speaker-independent models are presented in Table 5, depicting the obtained mean accuracy values for all classifiers.

Regarding the recognition results for Speaker 1, it is possible to verify that the BAG classifier produced the best recognition result (M=80.50) for the model trained with the data from all other speakers. Concerning Speaker 2, SVM achieved the best recognition accuracy (M=47.30) for the model trained with the data from the remaining speakers, however, it produced a better classification score for when the data from Speaker 4 was not yet included (M=49.00). Speaker 3 results share some similarities with the ones obtained from Speaker 2, with SVM also achieving the best recognition accuracy (M=43.40) for the model trained with the data from the remaining speakers but produced a better classification score for when the data from Speaker 4 was not yet included (M=45.30). Finally, for Speaker 4, BAG classifier produced the best recognition accuracy (M=81.80) for the model trained with the data from all other speakers.

**Figure 6:**
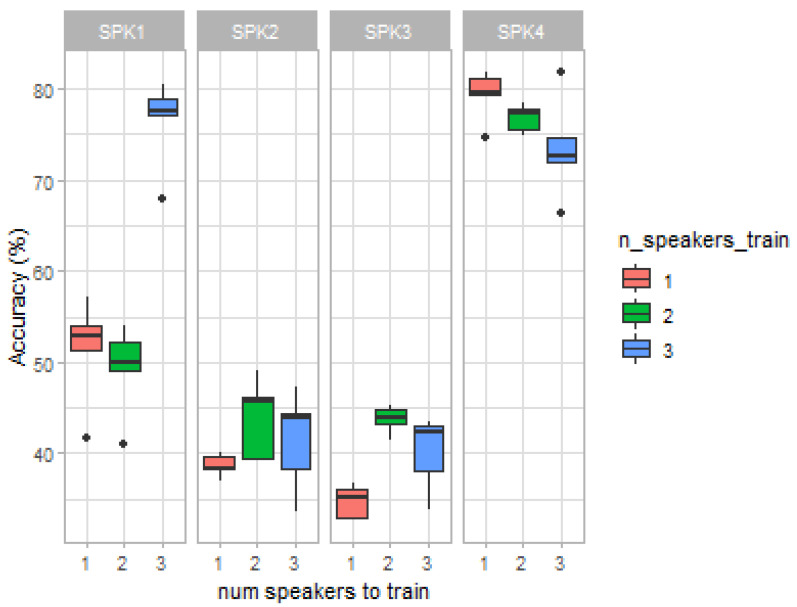
Study of speaker-independent models performance. Accuracy results, per speaker, when considering the remaining one, two, or three speakers for model training and subsequent classification).

Figure 6 allows further verifying the mentioned recognition similarities between two different groups of speakers (i.e., speakers 1 and 4, and speakers 2 and 3). Speakers 1 and 4 achieved higher recognition accuracies when the models were trained with data containing each other’s data, being their accuracy rates, with the models trained with all the remaining speakers’ data, superior to the ones achieved for speakers 2 and 3. However, for speakers 2 and 3, higher recognition accuracies were obtained when the models had not yet been fed with speakers 1 and 4 data.

## 5. Discussion

This work aimed to assert if, by resorting to FMCW radar, and expanding our preliminary work [13], three core aspects for SSI development could be tackled with this technology: (a) how capable is radar-based technology of successfully recognizing silent speech, (b) how discrepant can the results get when performing different acquisition sessions for the same participant), and (c) to what extent can inter-speaker models be created by resorting to this technology.

Concerning the per-speaker experiment, the average accuracy results of 84.50% using the BAG classifier and 88.30% using the LR classifier for both acquisition sessions translate into positive indications of FMCW radar-based technology SSR capabilities, particularly given that a set of thirteen words was considered. Through a careful analysis of the obtained results, it was possible to verify the positive impact that producing the words more consistently throughout the acquisition sessions can have in the establishment of more representative classification models. While the results were good for all speakers, this hints that such a simple instruction given to the speaker can potentially improve the recognition accuracies. Regarding the lower recognition accuracies for the words “L**em**bretes” (Reminders), sometimes recognized as “**Em**ail”, and “Li**g**ar” (Turn On), sometimes recognized as “Se**g**uinte” (Next), we believe that this may be due to some notable articulatory similarity, e.g., at the beginning or middle of the word that, at some elocution speeds and, eventually, as a consequence of coarticulatory effects, may turn their velocity dispersion patterns more similar.

Comparing the obtained recognition results with previous works for the same AAL corpus allowed verifying that radar-based SSR either obtained comparable or superior accuracy values. In [49], an accuracy of 75.00% is reported for sEMG, and in [45], accuracies of 71.40%, 72.60%, and 83.00% were obtained for Video, Depth, and UDS technologies, respectively. Further comparing the obtained results with the ones in the existing literature for several other technologies, although such comparisons may not be necessarily fair given different considered corpus and technologies’ nuances, allowed verifying that either comparable or superior results were also achieved. Two examples of such studies are the work by Sarmiento et al. [23], where the authors resort to EEG obtaining accuracies in the range of 67.78% and 72.67% for five syllables, and the work by Dash et al. [40], exploring Magnetoencephalography (MEG), and achieving an accuracy rate of 79.93% for five imagined phrases. Sun et al. [35] was capable of presenting an overall superior average recognition than the ones obtained in this study for a set of 20 commands. Nevertheless, the data considered resulted from asking the participants to over-articulate, something that is not considered in our work. Another representative study that managed to get a slightly superior recognition rate was the one by Kapur et al. [20], having achieved a 92.01% accuracy rate for a corpus of 15 words while resorting to sEMG.

Towards assessing the possibility of creating session-independent models from FMCW data, i.e., how a model created with data from one acquisition session can be used to perform recognition for another acquisition of the same speaker, a second research experiment ensued. Session recordings variability for the same participants is an aspect that limits several technologies considered towards SSR, typically requiring additional normalization algorithms across different sessions [27,28]. An analysis of the obtained results confirmed our initial hypothesis that whenever consistency is considered throughout the acquisition sessions it has a positive influence on the model’s performance. Such aspect is clear from the outcomes as speakers asked to attempt being consistent were the one with higher recognition rates in the per-speaker experiment and also achieved higher performance between sessions.

Further comparing the acquired results with those presented in the literature for other technologies would be ideal. However, after an extensive review (Table 1), and to the best of our knowledge, most of the studies that mention session-independence aspects either focus their efforts on researching and developing normalization methods for tackling data variance between different acquisition sessions for the same participants [27,28] or highlight it as an aspect to explore in future work [32].

The final study’s assessment—speaker-independence—aimed to assess the extent to which it was possible to create representative speaker-independent models by resorting to FMCW radar-based technology. By analyzing the obtained accuracy values, what stands out the most is that the results are significantly better when data from the two speakers asked to be consistent throughout the acquisition sessions (SPK1 and SPK4) is considered. Whenever one of these speakers is included in the training data, the accuracy for the other shows a strong improvement. Therefore, consistency seems to also play a pivotal role, here, enhancing the importance of particular patterns of articulation that are similar for these two speakers. Nevertheless, the nature of this advantage is yet to be established, particularly if it extends to more speakers observing a principle of consistency during the acquisition.

Another aspect worth noting concerns the higher (although to a smaller extent) recognition accuracies achieved for speakers 2 and 3 when the models had not yet been fed with data from speakers 1 and 4. The impact of the data from speaker 1 and 4 on the performance of models created considering speakers 2 and 3 may be sourced in a wide variety of factors: (1) first, we know that speakers 1 and 4 were instructed to be consistent and, therefore, it is conceivable that their data patterns have some degree of similarity and consistency, making the presence of one of them very favorable for the other, as our results show; (2) this consistency probably makes speakers 1 and 4 diverge more from speakers 2 and 3 and, thus, when these two ’kinds’ of data are mixed, the resulting model is less capable of dealing with each of these latter speakers; and (3) naturally, this is potentiated by the moderate amount of speakers and data considered. In this context, future tests with a wider range of speakers may help clarify this aspect.

Still, the results obtained in this first experiment with speaker-independent models for FMCW radar are quite positive as, for the models created with three of the speakers, the worst case yielded 43.40% accuracy for a corpus of 13 words.

The possibility of creating speaker-independent models is an aspect that has been highly desired towards the development and integration of SSI in more ecological settings, allowing speakers to use the systems without requiring them to have previously contributed to the data used to create the model. Several studies, exploring different technologies (e.g., Video, US, sEMG, and MEG), have already stated the importance of considering such speaker-independent models; however, most either achieve poor recognition results or solely mention the topic and leave such consideration for future endeavors [14,20,30,31,32,36,38,40]. In this regard, two studies are worth mentioning. Sun et al. [35] explored video technology towards SSR and achieved an accuracy of 95.40% for 20 limited context-usage commands with over-articulation of the lips in speaker-independent settings, while Petridis et al. [38], also having explored video technology, achieved results ranging the 70.00% mark for two different corpus of respectively ten digits and ten phrases. One aspect, however, that highly contributes towards this technology’s capability of creating speaker-independent models is that there already exist large volumes of data for several different speakers, something that does not happen for many other technologies. Nevertheless, video technology still remains dependent on ambient lighting and privacy concerns. Besides video technology, several other studies also mention how relevant achieving speaker-independent models would be but rarely explore it, typically leaving such consideration for future work [14,20,30,31,32].

Besides the promising results already presented, there are, nevertheless, some limitations of the present work that are worth noting. Regarding the acquisition sessions, the distance between the participant and the radar, although not being enforced as in similar studies in the literature, see, e.g., in [8], was kept at around 15cm with minor variations around this value. Although being less demanding and not obligating the speakers to be attached to the technology or remain immobile during the acquisitions, as it frequently happens in other technologies (e.g., US, EEG, and MEG), this is not yet the full extent of the capabilities we envisage for the technology to serve the considered scenarios.

Another limiting aspect resides in the fact that the considered corpus, although already comprising a word set comparable to the existing literature, contains inputs suited for specific interaction scenarios. Although capable of serving multiple domains, considering different interaction contexts usually requires different types of commands.

Finally, one last limitation concerns the number of speakers considered for acquisition. Promising results were achieved in all carried experiments with the four considered speakers. However, considering a higher number of speakers would allow establishing more generalized models by taking into account different individuals’ anatomies and articulatory idiosyncrasies.

## 6. Conclusions

This paper proposes and demonstrates the consideration of a FMCW radar board to assess the plausibility of contactless radar-based technology towards SSR. Besides demonstrating its SSR capabilities, additional experiments were also performed to verify the possibility of creating session and speaker-independent models.

Regarding the per-session speaker experiment, based on velocity dispersion features, several classification models were trained and were subsequently capable of producing average recognition accuracies as good as 84.50% for the first acquisition session and 88.30% for the second one. Accuracies of 81.79% and 71.79% were also obtained for the session-independence experiment, suggesting that this technology may be resilient to variations in the recording data across different acquisition sessions for the same speakers. For the final carried experiment, Speaker-Independence, focusing on the possibility of training speaker-independent models, recognition accuracies as high as 80.50% and 81.80% were also achieved.

The obtained results, along with the inherent advantages of contactless radar-based technology (e.g., its non-invasive and privacy-preserving nature, its portability, and robustness against lighting conditions and environment noise), establish promising grounds for further exploring and more frequently considering this technology towards SSI development purposes.

Regarding future work, the team’s focus will be on acquiring a larger amount of radar data from a wider range of speakers. This would allow us to consider other well-known classification models that require larger volumes of data (e.g., artificial neural networks (ANN) and convolutional neural networks (CNN)) and, most importantly, understand the impact that data from several participants has in the creation of the so needed speaker-independent models. Besides this central focus, the influence of speaker-to-radar distance and head orientation are other aspects that are deserving our attention. Initial assessments with different head orientations were already performed and allowed verifying that data from different orientations, when considered together with the frontal one, can improve recognition results, as the models learn how to more easily discern the word classes in which there is articulatory ambiguity. However, more experiments still need to be carried out including, e.g., multi-radar settings.

## Figures and Tables

**Table 2:** Corpus considered for all radar data acquisition sessions, containing a total of thirteen European Portuguese words.

Ajuda	(help)	Anterior	(previous)
Calendário	(calendar)	Contactos	(contacts)
Email	(email)	Familia	(family)
Fotografias	(photographs)	Lembretes	(reminders)
Ligar	(turn on)	Mensagens	(messages)
Pesquisar	(search)	Seguinte	(next)
Vídeos	(videos)		

**Table 3:** Mean (M), standard deviation (SD), and maximum (max) accuracy value for a specific k-fold iteration, and average recognition scores per speaker obtained for the different classifiers. For each column the highest recognition score is presented in bold face. All metrics were calculated and present values that are respective to a particular acquisition session.

Classif.	M		SD		Max		SPK1		SPK2		SPK3		SPK4	
	1st	2nd	1st	2nd	1st	2nd	1st	2nd	1st	2nd	1st	2nd	1st	2nd
RF	83.50	86.00	7.30	3.70	93.60	91.00	88.10	88.20	**76.00**	-	80.60	82.00	89.40	88.00
BAG	**84.50**	86.20	8.70	4.00	95.50	91.70	**91.50**	87.60	74.00	-	**82.20**	81.90	90.10	89.10
LDA	81.40	86.30	9.80	5.40	94.20	93.60	88.70	89.70	71.70	-	74.50	79.80	90.80	89.60
LR	83.20	**88.30**	10.20	4.70	**96.10**	**96.10**	**91.50**	**92.20**	74.40	-	75.00	**83.10**	**92.20**	**89.80**
SVM	80.70	86.10	10.50	5.20	94.20	93.60	88.80	89.10	70.80	-	72.20	79.50	90.90	89.70

**Table 4:** Mean accuracy values, produced by all classifiers, for the Intra-Speaker experiment. Acquired data from the first session (session 1) was used for training, while data from the second session (session 2) was considered for testing. Relative variances between session-independent results and per-session results are also presented for each speaker. The highest values in each column are highlighted using bold face.

	SPK 1 (S1) -> (S2)	R.Variance (S1)	R.Variance (S2)	SPK 3 (S1) -> (S2)	R.Variance (S1)	R.Variance (S2)	SPK 4 (S1) -> (S2)	R.Variance (S1)	R.Variance (S2)
RF	74.10	**14.00 (88.10)**	14.10 (88.20)	**67.95**	12.65 (80.60)	14.05 (82.00)	65.90	23.50 (89.40)	22.10 (88.00)
BAG	**81.79**	9.71 (91.50)	5.81 (87.60)	65.77	**16.43 (82.20)**	**16.13 (81.90)**	**71.79**	18.31 (90.10)	17.31 (89.10)
LDA	75.38	13.32 (88.70)	**14.32 (89.70)**	63.97	10.53 (74.50)	15.83 (79.80)	66.15	24.65 (90.80)	23.45 (89.60)
LR	78.08	13.42 (91.50)	14.12 (92.20)	67.17	7.83 (75.00)	15.93 (83.10)	63.33	**28.87 (92.20)**	**26.47 (89.80)**
SVM	76.53	12.27 (88.80)	12.57 (89.10)	66.28	5.92 (72.20)	13.22 (79.50)	68.46	22.44 (90.90)	21.24 (89.70)

**Table 5:** Mean accuracy values, produced by all classifiers, for the Inter-Speaker experiment. The data belonging to each speaker were left out for testing, while the models considered for recognition were trained with the data belonging to the remaining speakers. The results were obtained considering the data for the first acquisition session, for each speaker. The highest mean accuracy obtained for each experiment is shown in bold face.

	SPK1			SPK2		
**Model**	**SPK2**	**SPK2 + SPK3**	**SPK2 + SPK3 + SPK4**	**SPK1**	**SPK1 + SPK3**	**SPK1 + SPK3 + SPK4**
RF	51.40	49.00	79.00	**40.10**	45.80	44.30
BAG	54.10	**54.00**	**80.50**	36.90	39.50	38.20
LDA	41.70	40.90	77.18	38.20	39.30	33.60
LR	52.80	50.00	67.90	38.30	46.20	43.80
SVM	**57.20**	52.20	77.60	39.70	**49.00**	**47.30**
	**SPK3**			**SPK4**		
**Model**	**SPK1**	**SPK1 + SPK2**	**SPK1 + SPK2 + SPK4**	**SPK1**	**SPK1 + SPK2**	**SPK1 + SPK2 + SPK3**
RF	32.60	43.80	42.30	74.70	75.60	74.70
BAG	**36.80**	44.70	42.90	**81.90**	77.80	**81.80**
LDA	32.90	43.30	33.80	79.40	77.40	72.60
LR	36.00	41.50	38.00	79.50	74.90	66.40
SVM	35.10	**45.30**	**43.40**	81.20	**78.50**	71.90

## Data Availability

The data presented in this study are available on request from the corresponding author. The data are not publicly available due to privacy concerns.
